# Can Postoperative CT Imaging in Spine Surgery Be Replaced by Intraoperative 3D Rotation With the C-Arm?: Results of a Prospective Single Center Cohort Study

**DOI:** 10.3389/fsurg.2021.692189

**Published:** 2021-07-14

**Authors:** Mohammed Banat, Johannes Wach, Abdallah Salemdawod, Lisa Domurath, Jasmin Scorzin, Hartmut Vatter

**Affiliations:** Department of Neurosurgery, University of Bonn, Bonn, Germany

**Keywords:** spinal surgery, patient safety, screw accuracy, technologies in spinal surgery, intraoperative navigation

## Abstract

**Objective:** Dorsal instrumentation of the spine is an established treatment option for a range of spinal pathologies. Intraoperative fluoroscopy connected with navigation minimize the risk of incorrect screws placement. In several cases, post-operative CT scans are needed to verify possible mismatches. In this study, we evaluated the efficacy of 3D intraoperative fluoroscopy as compared to post-operative CT and the need of post-operative CT.

**Methods:** We conducted a prospective single-center cohort study, 94 patients were included. The screws were implanted using 3D rotation with C-arm and navigation system. The definitive position of the screws was verified by a post-operative CT scan. Finally, we compared the discrepance between intraoperative imaging and post-operative CT scan using Rampersaud-grade (A-D).

**Results:** 607 screws in 94 patients were included. Some 3% of the screws had to be replaced immediately intraoperative due to inadequate position with lateral or medial trajectory. An A-score was achieved for 85.5% of the 3D controlled screws and 87% of the post-operative CT. A B-score was found in 11.5% of either groups. In the 3D group a C-score was achieved for 2.5% and in the CT group for 0.8%. A D-score was found in 0.5% of the screws in both groups, *p* = 0.45. Only a mismatch of 3% could be detected for the intraoperative and post-operative imaging results.

**Conclusion:** Our study data shows that the placement of screws using the 3D rotation and navigation tool is safe and accurate. There were no relevant mismatches between intraoperative images and the post-operative CT.

## Introduction

Dorsal instrumentation of the spine is an established treatment option for a range of spinal pathologies, including instable trauma, degenerative diseases, deformity of the spine, neoplasia and infection (1–3). To this day, the “free-hand” technique using fluoroscopy is the approach most commonly used (4). The use of intraoperative fluoroscopy during the operation in combination with coupled navigation system reduces the risk of incorrect placement of the screws (5–8). Nevertheless, this technique is used more often in academic and scientific than in community institutions (9), and the important question of which method and kind of instrumentation are better, is the recent subject of controversial discussion in the literature. Therefore, the main aim of this study was to evaluate the effectiveness and usefulness of the intraoperative 3D fluoroscopy with C-arm (in combination with coupled navigation system) for placing screws and to consider screw placement during the operation as compared with post-operative control CT scans and the necessity of post-operative CT.

## Methods

### Patient Selection and Inclusion Criteria

This prospective single-center cohort study analyzes patients with dorsal instrumentation at our Institution (Level 1 Center for Spine surgery) between December 2017 and January 2019. Patients were included if they received dorsal instrumentation to treat instable fracture, infection of the spine as spondylodiscitis, tumor with bone destruction, or degenerative disease. Data were documented and analyzed for age, ASA, BMI, blood loss, pathologies, complication and duration of surgery. Furthermore, screws position were documented.

Inclusion criteria were instability with bone destruction owing to fracture after trauma, tumor, spondylodiscitis, adult degenerative deformity, and consent of the patient. All patients were over 18 years of age. Exclusion criteria were emergency surgery at night, lack of interest in the study, and when the patients had been operated using a conventional approach (free-hand technique). The investigation was approved by the local ethics committee (protocol no. 350/17). The patients/participants provided their written informed consent to participate in this study.

**Figure 1:**
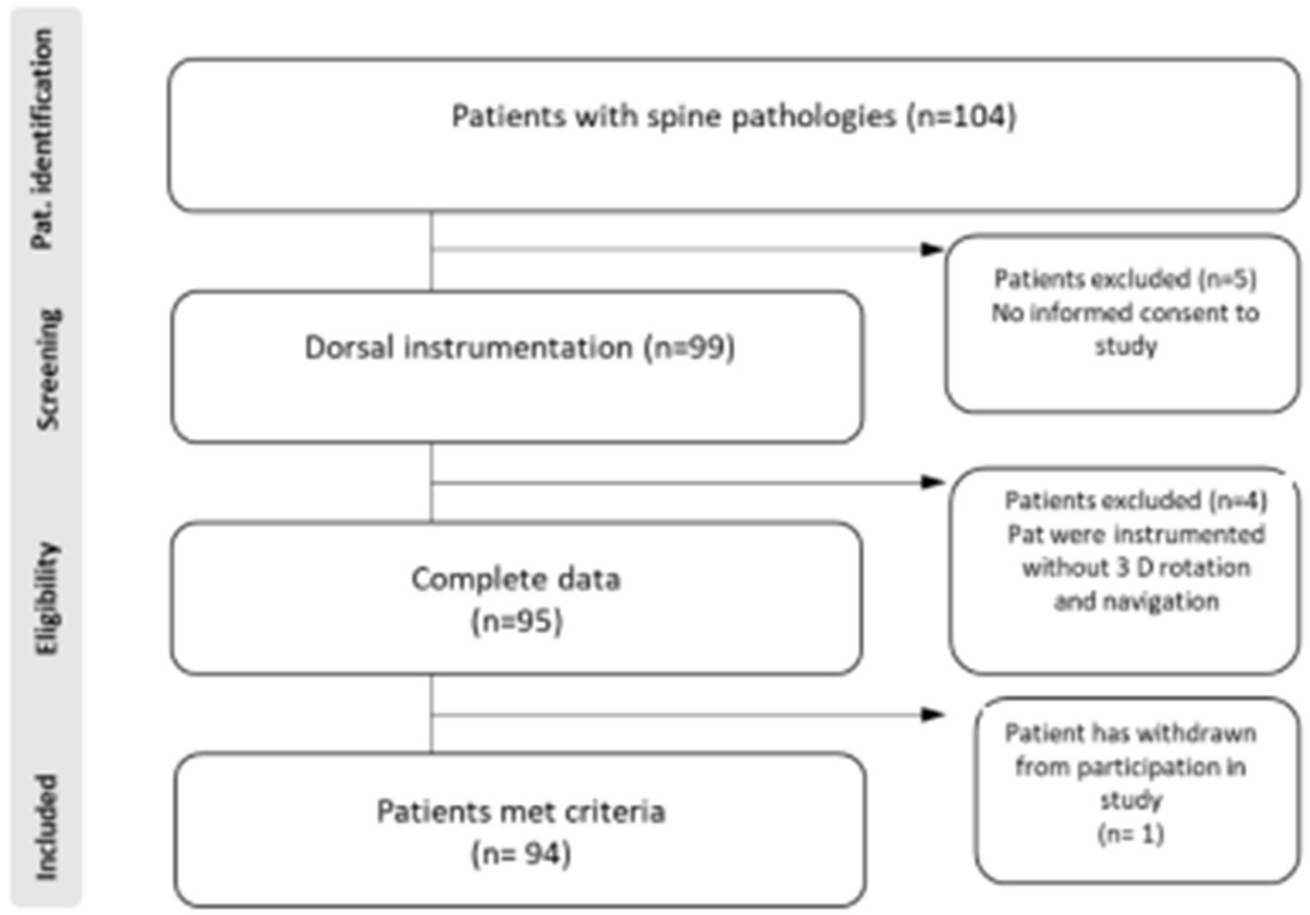
Flowchart of patient inclusion criteria.

**Figure 2:**
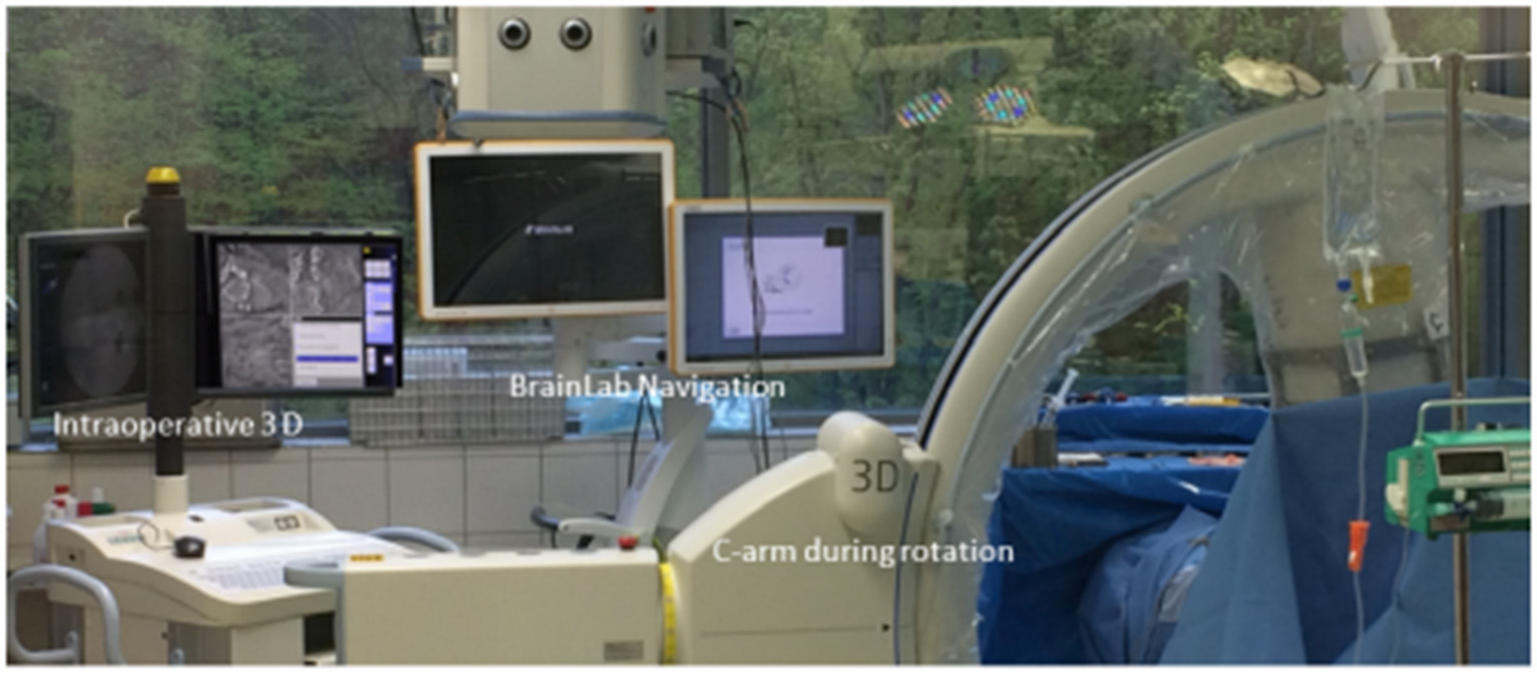
Live Foto from the intraoperative room at our institution during the 3D rotation.

**Figure 3:**
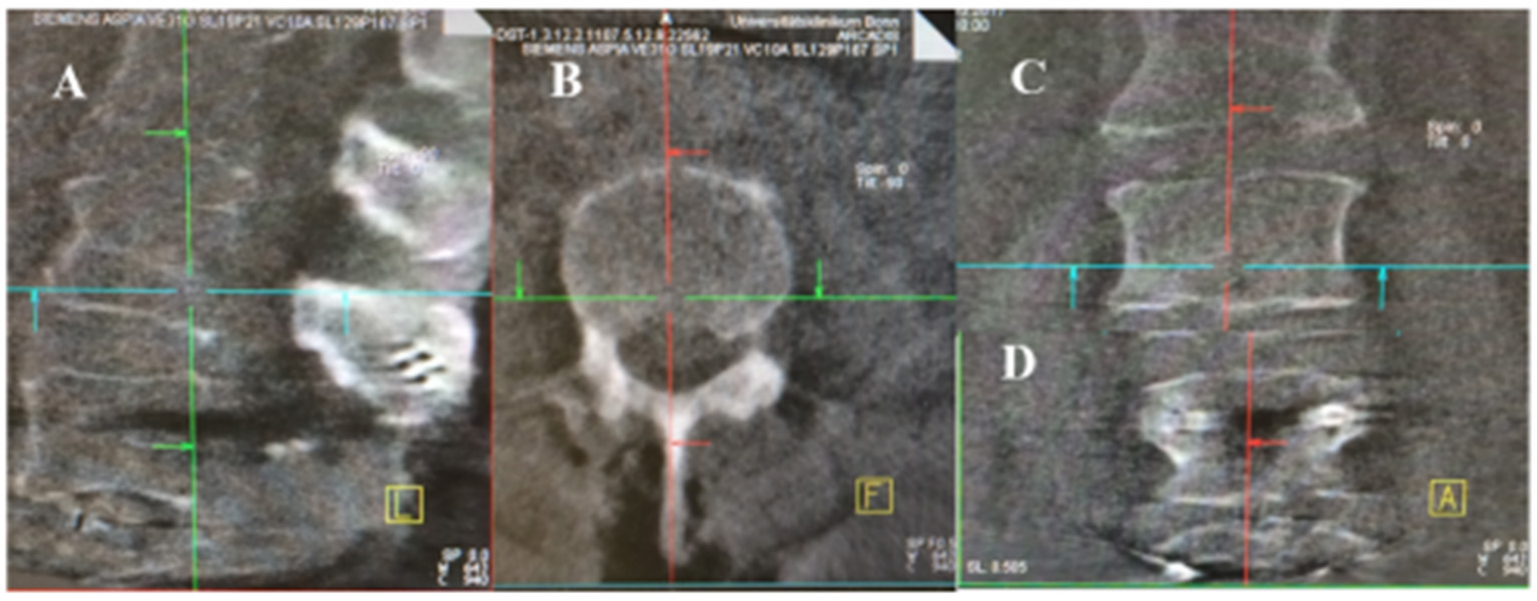
Intraoperative 3D Rotation with C-arm. **(A–C)** the first scan bevor screws implantation (**A**: sagittal, **B**: axial, **C**: coronar). **(D)** after screws implantation in coronar sequence.

In total, 94 patients met the study inclusions criteria (Figure 1). Intraoperative images were carried out using a 3D C-arm (Firm Siemens-Arcadis Orbic) with a coupled navigation system (Vector Vision, Firm BrainLab). During the scan the medical personal have to leave the operation room (Figure 2). There were two scans performed, the first before and the second scan after placing the screws (Figure 3). Postoperative surgical related complications and reasons for revision operation were determined and further analyzed. All operations were performed by three neurosurgeons at our department to eliminate each possible bias associated with a surgeon's expertise and experience.

### Radiological Evaluation

The definitive position of the screws was assessed by a post-operative CT scan. Therefore, we could check mismatches between position results of intraoperative (3D) assessment after screws implantation and post-operative CT.

**Figure 4:**
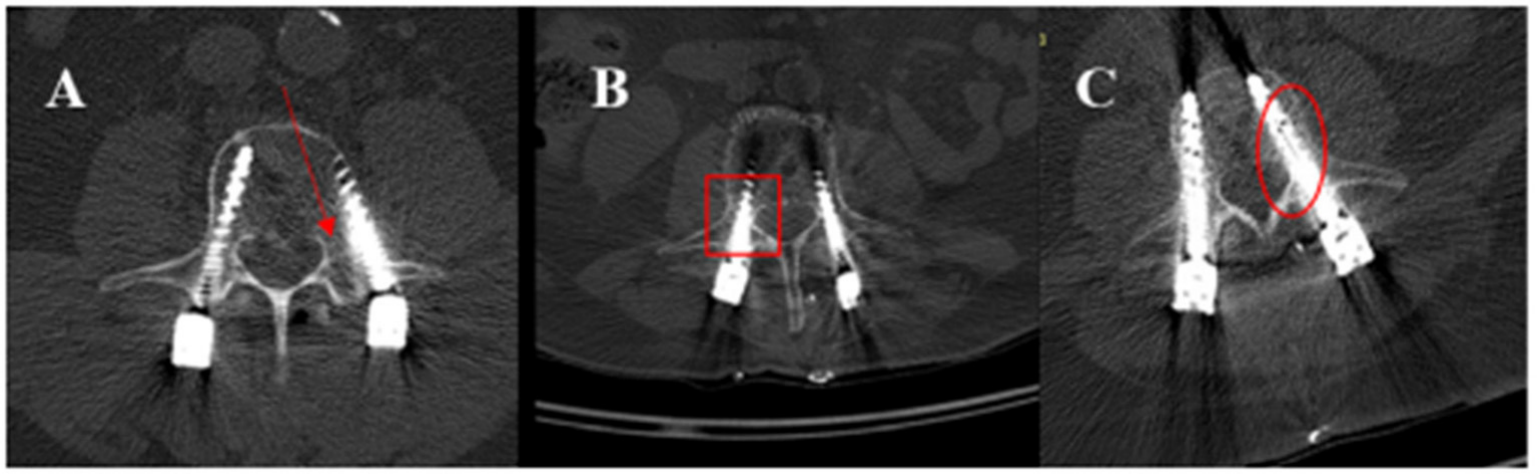
Post-operative CT illustrates the position of the screws according to Rampersaud graduation. **(A)** The right pedicel demonstrates a grade A position; the screw in the left pedicel shows a D grade (with the red arrow marked). **(B)** shows the screw in the right pedicel with Grad B pedicle wall breach < 2 mm- (marked with a square). **(C)** illustrates a screw position in the left pedicel with grade C – the wall of pedicle breach equal to 2–4 mm- (with the circle marked) without neurological symptoms. In these cases, no revision is needed.

Independent neuroradiologist analyzed the post-operative CT control and the screw position. The grade of Rampersaud (10) was deployed to further classify positioning of the implanted screws: “(A: completely within the pedicle; B: pedicle wall breach < 2 mm; C: pedicle wall breach equal to 2–4 mm; and D: pedicle wall breach more than 4 mm)” (Figure 4).

### Statistical Analysis

Data analyses were performed using the SPSS computer software package v.25 (IBM Corp., Armonk, New York, United States) for Windows. Categorical variables and intergroup comparisons were performed using Student's *t*-test for unpaired samples, using Fisher's exact test and chi-squared test (two-sided). Results with *p* < 0.05 were considered statistically significant. Data were described as means and standard deviation (SD) and frequency (*n*).

### Surgical Technique and Procedure

**Figure 5:**
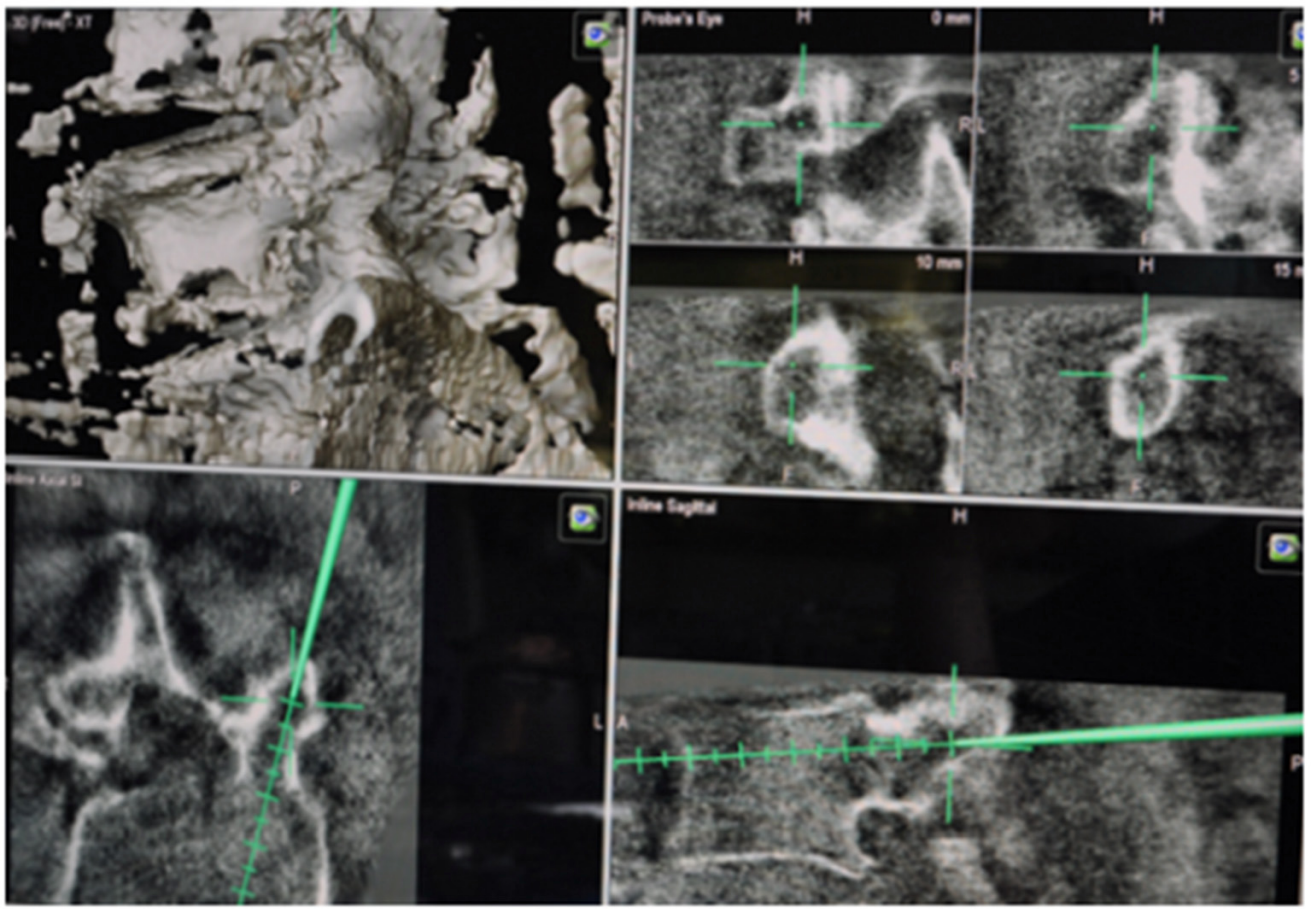
Intraoperative navigation with BrainLab system. This shows the trajectories of screws transpedicular.

All of surgical procedures and operation were performed under general anesthesia. The prone position was the standard position. We used a radiolucent carbon table. A midline posterior approach was taken at the levels of the segments to be instrumented; the paraspinal muscles were removed with monopolar electrocautery forceps, the anatomic entry points for the screws were marked. An isocentric 3D C-arm connected to our navigation system (BrainLab) was used for spinal navigation. Before beginning of 3D scan, the carbon reference array was fixated to the central spinous process of the levels to be instrumented and the first scan was performed. During the 3D fluoroscopic scan left all of the medical personnel the operating room (Figure 2). Screws were implanted by using the navigation system alone (Figure 5). Cannulated drill guides and cannulated screws were integrated with the help of a K-wire. After drilling of the bone, a K-sonde was then used to confirm that the screws have a correct trajectory. Finally, a second 3D rotation fluoroscopic scan was routinely executed after inserting all screws to check that the screws were accurately placed, if a screw was misplaced, this was corrected directly during the same session, as already described. After new correction a third and finally scan was performed.

### Postoperative Management

Postoperatively, all of patients received early post-operative mobilization with physiotherapy on the first day after surgery. Wound drains were removed on post-operative day 2–3, sutures on post-operative day 7–10. Control CT scans were acquired directly on the same day after the operation.

## Results

A total of documented 607 screws in 94 patients were included in the Analysis. Table 1 shows the baseline patients data. There was no relevant significant difference in the female-to-male ratio or ASA Score. The division of the conditions underlying spinal instability is illustrated in Table 1, with the indications for the operation. The term “another location” involves the cervicothoracic, thoracolumbar or lumbosacral transition.

**Table 1:** Baseline data.

**Total (*N* = 94)**		**%**
**Age y/o**	70.2 (55.0–78.0)	
**Gender**		
Female	40	42.5%
Male	54	57.5%
**ASA**		
1&2	45	47.87%
3&4	49	52.13%
**BMI (mean ± SD)**	27 (SD ± 3)	
**Localization**		
Cervical	12	12.8%
Thoracic	17	18.1%
Lumbar	54	57.4%
Another location	11	11.7%
**Operating time (min.)**	269.5 (201.0–382.5)	
**Blood loss (mL)**	900 (500–1,800)	
**Scan time (min.)**	3 (SD ± 2.2)	
**Complication**		
Neurological worsening	0	0
Death	2	2%
Wound dehiscent/infection	3	3%
**Pathologies**		
Fracture	21	22.3%
Degenerative	30	31.9%
Tumor	22	23.4%
Infection	21	22.3%

*SD, Standard deviation; ASA, American society score; BM, Body Mass Index*.

All of the screws were implanted and inserted using the navigation system coupled with 3D C-arm (Figures 2, 4). After definitive implantation of the screws, a finally 3D rotation study was performed to check the screws position. Here, 3% (*n* = 20) of the screws had to be replaced directly due to inadequate positioning, the screws showed lateral (outside of the bone) or median projection of the pedicle with contact to the neural structure.

Overall post-operatively, 85.5% (*n* = 520) of the 3D-controlled screws and 87% (*n* = 530) of those on the post-operative CT scan achieved an A-score (optimal position). A B-score was found in 11.5% (*n* = 70) of both groups (good position). In the 3D group 2.5% (*n* = 15) and in the CT group 0.8% (*n* = 5) achieved a C-score ***(P = 0.03)***. A D-score was found in 0.5% (*n* = 2) of the screws in both groups. Spearman's correlation analysis with regard to screw positioning in intraoperative 3D scan and post-operative CT scan revealed a strong correlation (Spearman's rho: 0.935, *p* < 0.001). A mismatch of only 3% (*n* = 20) of the inserted screws was detected between the intraoperative and post-operative imaging results (Table 2).

**Table 2:** Graduation and comparisons using chi-squared test (two-sided).

**Screws (*n* = 607)**	**3D % (*n*)**	**Postop CT % (*n*)**	***P*-value**
A-grade	85.5% (520)	87% (530)	0.45
B-grade	11.5% (70)	11.5% (70)	0.98
C-grade	2.5 % (15)	0.8 % (5)	0.03^*^
D-grade	0.4% (2)	0.4% (2)	0.99

* *Statistical significance*.

## Discussion

During the recent 10 years surgical expertise, technical progress, and digitalization have come to play an increasingly important central role in spinal surgery and treatment of patients. The aim of this study was to evaluate the role and importance of an intraoperative imaging guidance system (3D fluoroscopy with C-arm) for placing screws in spinal surgery and to evaluate the necessary of post-operative CT after surgery. There are certainly enough works in the world literature to prove the usefulness of the technique we use, but there are limitations about the need for post-operative imaging. In our cohort single center study at our department, 94 patients with a total of 607 screws were included and systematic analyzed. We found that 97.5% of the implanted screws were positioned with high accuracy using the 3D rotation coupled with the navigation (Grade A and B). There was no relevant significant difference between the guidance system 3D fluoroscopy and the post-operative CT scan. The rate of incorrect position of screws with a grade of C and D was 3%. Sometimes, the suboptimal positioning was recognizable by the surgeon directly during operation but the screw placement was not new replaced owing to the mild or poorly quality of the bone. These cases in our series involved osteoporosis associated fracture (3 patients) and, in the rest, spondylodiscitis with destruction of the vertebra. We observed no CSF fistula due to misplaced screws in the present study. All of the patients, who were surgically treated for a lumbar pathology underwent surgery without neuromonitoring. Further frustrating manipulation in this setting tended to worsen with impaired lower bone quality. On the other hand, any malpositioned screw that requires revision may extend the operating time, increase a high dose of radiation, cause more blood loss, or even lead to neurologic and systemic complications during or after surgery (11–15). For spine surgeons a “clinically acceptable” screw position score is A or B (16). According to our data, the finally position of the screws and estimation in the 3D rotation was not inferior to the post-operative control CT scan; therefore, a post-operative CT scan at our department is not necessary for the future after dorsal instrumentation. In our study the percentage rate of 3% for pedicle wall perforation (lateral or medial) appears to be well within the range of 1.2–41.0% widely reported in the worldwide literature (10, 17–19). The study group Staartjes et al. showed in meta-analysis and systematic review of pedicle screw revision in 2018 that: “only 14 of 37 studies (38%) reported accurately on intraoperative screw revisions, intraoperative revisions were equally common in the navigation and free-hand technique groups (*P* = 0.64); however, the comparison of navigation and free-hand did show significant heterogeneity (*P* < 0.001).” The corresponding funnel plot suggests some reporting bias (20).

Considering the low radiation exposure for medical personal and for the patients, there is a clearly low exposure with the use of 3D intraoperative, the reported radiation exposure time for placing a pedicle screw varies in the literature from 3.4 to 66 s per screw (21–23); our scan time intraoperative is in the range.

A current discussion in spine society is the use of O-arm instead of the C-arm, however, taking into consideration that O-arm is not widely available in all medical centers, our results show that C-arm still represents a comparable accuracy and therefore considered as an adequate alternative for O-arm.

## Limitation

The main limitation of our work is that we reported results of a single-center cohort study. Additionally, the expertise in the use of navigation combined with the 3D rotation in our center is still limited by the short period since the introduction of this system in our center. Another limitation is the small group of patients within a short time.

## Conclusion

Our study data shows that the placement of screws using a 3D rotation coupled with the navigation tool is effective and accurate. There were no significant mismatches between the intraoperative fluoroscopy images and the post-operative control CT scans. Therefore, our study suggests a reevaluation of the necessity of post-operative CT scans.

## Data Availability Statement

The original contributions presented in the study are included in the article/supplementary material, further inquiries can be directed to the corresponding author.

## Ethics Statement

All of the procedures performed were in line with the ethical standards of our institutional and national research committee (Ethic committee of the Rheinische Friedrich Wilhelms University Bonn) and with the 1964 Helsinki declaration and its later amendments or comparable ethical standards. The local ethics committee –University of Bonn- (Protocol no. 350/17), approved the investigation of this study. **Study design:** Prospective clinical cohort single center study. The patients/participants provided their written informed consent to participate in this study.

## Author Contributions

MB: first drafting, conceived, designed and performed the study, and wrote the manuscript. JW, AS, and JS: critical review of the manuscript. LD: study design and statistical analysis. HV: critical review of the manuscript and analysis and interpretation of data. The final drafting was critically revised and considered by all authors.

## Conflict of Interest

The authors declare that the research was conducted in the absence of any commercial or financial relationships that could be construed as a potential conflict of interest.
